# Development and validation of a deep-broad ensemble model for early detection of Alzheimer's disease

**DOI:** 10.3389/fnins.2023.1137557

**Published:** 2023-07-11

**Authors:** Peixian Ma, Jing Wang, Zhiguo Zhou, C. L. Philip Chen, The Alzheimer's Disease Neuroimaging Initiative, Junwei Duan

**Affiliations:** ^1^College of Information Science and Technology, Jinan University, Guangzhou, China; ^2^College of Computer Science, Guangdong Polytechnic Normal University, Guangzhou, China; ^3^School of Integrated Circuits and Electronics, Beijing Institute of Technology, Beijing, China; ^4^School of Computer Science and Engineering, South China University of Technology, Guangzhou, China; ^5^Steering Committee of Alzheimer's Disease Neuroimaging Initiative, Bethesda, MD, United States; ^6^Guangdong Provincial Key Laboratory of Traditional Chinese Medicine Informatization, Guangzhou, China

**Keywords:** deep-broad ensemble model, Alzheimer's disease, early detection, MRI, validation, efficiency

## Abstract

**Introduction:**

Alzheimer's disease (AD) is a chronic neurodegenerative disease of the brain that has attracted wide attention in the world. The diagnosis of Alzheimer's disease is faced with the difficulties of insufficient manpower and great difficulty. With the intervention of artificial intelligence, deep learning methods are widely used to assist clinicians in the early recognition of Alzheimer's disease. And a series of methods based on data input with different dimensions have been proposed. However, traditional deep learning models rely on expensive hardware resources and consume a lot of training time, and may fall into the dilemma of local optima.

**Methods:**

In recent years, broad learning system (BLS) has provided researchers with new research ideas. Based on the three-dimensional residual convolution module and BLS, a novel broad-deep ensemble model based on BLS is proposed for the early detection of Alzheimer's disease. The Alzheimer's Disease Neuroimaging Initiative (ADNI) MRI image dataset is used to train the model and then we compare the performance of proposed model with previous work and clinicians' diagnosis.

**Results:**

The result of experiments demonstrate that the broad-deep ensemble model is superior to previously proposed related works, including 3D-ResNet and VoxCNN, in accuracy, sensitivity, specificity and F1.

**Discussion:**

The proposed broad-deep ensemble model is effective for early detection of Alzheimer's disease. In addition, the proposed model does not need the pre-training process of its depth module, which greatly reduces the training time and hardware dependence.

## 1. Introduction

Alzheimer's disease (AD) is a chronic neurodegenerative disease of the brain that develops insidiously. People diagnosed with Alzheimer's will suffer from the disease for remaining lifespan (Todd et al., 2013; Weiner et al., 2013; Fink et al., 2020). The main symptoms of AD includes memory impairment, executive dysfunction, aphasia, impairment of visuospatial skills and so on, and the etiology remains unknown (Mayeux and Sano, 1999; Mimura and Yano, 2006). Thus, Millions of people around the world suffer from Alzheimer's disease. The long-term treatment of these patients consumes huge medical resources and costs (Cummings and Cole, 2002; Scheltens et al., 2016). The diagnosis of AD can be divided into three main types: AD (Alzheimer's disease), MCI (Mild Cognitive Impairment), NC (Normal Control).

Radiographic images are important in medical diagnosis of Alzheimer's disease. These include positron emission tomography (PET), magnetic resonance imaging (MRI), computed tomography (CT) and so on (Prince and Links, 2006; Doi, 2007; Johnson et al., 2012). Due to low cost and high efficiency, MRI imaging play an important role in diagnosing AD related pathological brain changes and researching (Jack et al., 1999). The understanding of the pathological information provided by these radiographic images depends on the knowledge and experience of the front-line clinicians. As the number of professional medical staff is far less than the actual patient treatment needs, they can not timely diagnose some early hidden symptoms of Alzheimer's disease. At the same time, the imbalance of medical resources also leads to the inability of patients in rural areas to obtain effective early diagnosis locally for follow-up treatment.

Currently, with the proposal of VGG (Simonyan and Zisserman, 2014), ResNet (He et al., 2016), ResNest (Zhang et al., 2022), ResNext (Xie et al., 2017) and a series of deep neural networks, many medical and artificial intelligence researchers used these model to conduct corresponding training on radiographic images. The popularity of high-performance hardware maked it possible to deploy these frameworks in some large hospitals and enable medical departments to actually use these methods to assist physicians in clinical diagnosis and reduce patient care costs. Due to the complex 3-Dimensional (3D) spatial feature of radiographic images, which is extremely different from the traditional 2-Dimensional (2D) images, a variety of model were proposed based on different inputs. Compared to 2D-input models, 3D-input models get more structure information from data obtained by continuous scanning, thus they can extract more complex three-dimensional spital feature. Consequently, in most application scenarios, 3D-input models performs better than the former in recognition tasks. These methods can be divided into 2D deep learning method and 3D deep learning method. The 2D method mainly divides the original medical image into multiple slices on a specific axis and then inputs them into the classical convolutional neural network for training. However, the 2D method cannot learn the correlation feature between these slices, so the model performance is limited. The 3D method directly input the original image into the 3D improved convolutional neural network for training, in order to learn more comprehensive feature information and make up for the above defects.

However, deep models contained a large number of hyperparameters, which required huge hardware resources. The gradient descent method is also prone to fall into the optimal solution, leading to the failure of weight. Researchers need to find a quick and effective way to solve this problem. In recent years, on the basis of Random Vector Functional-Link Neural Network (RVFLNN) (Pao et al., 1994) and Single-layer Linear Feedforward Network (SLFN) (Sanger, 1989), Chen et al. proposed the Broad Learning System (BLS) (Chen and Liu, 2017a,b) and proved its approximation. BLS showed good accuracy and excellent calculation speed in various classification tasks.

Therefore, on the basis of BLS, we try to combine it with deep learning to establish a depth-broad ensemble model. The 3D deep convolution module will enable the model to have the capacity to initially extract features of 3D inputs, while the broad learning module, as a key part of feature fitting, greatly reduces the resource consumption of the model and can maintain a good performance. While Alzheimer's image recognition is a emblematic 3D image processing task, it has had a profound influence on medicine and computer science. There have been a lot of research on the application of depth model in this aspect. Applying our proposed depth-broad ensemble model to the early detetion of Alzheimer's disease will help drive technological innovation, reduce the cost of future applications, and better facilitate the adoption of machine learning technologies in this field.

In this study, we proposed an improved deep-broad ensemble model for the detection of AD. This model combined the 3D extraction capability with the fast operation speed and low dependence on hardware. It firstly extracted spatial features of different levels of images, and then fused multi-level features based on a novel BLS to get better classification results. We applied this model to the task of MRI image recognition in Alzheimer's disease and compared it with some previous work and the work of radiology readers. Experimental results demonstrate that the proposed model has excellent accuracy and computational efficiency.

The main contributions of our study for the early detection of AD can be reported as follows:

1. We constructed a novel deep-broad ensemble model based on 3D residual convolution module and Broad Learning System.
2. The proposed model outperforms previous single deep models, and has higher training efficiency, less dependence in hardwares.
3. There is no need to pre-train the deep modules of the proposed, which greatly reduces the training time.

## 2. Related works

Early detection of Alzheimer's disease is a chronic and significant research topic in computer science area. At present, a large number of computer-aided early detection methods for Alzheimer's disease have been developed. According to the dimension division of input data, related works can be divided into two-dimensional input-based research methods and three-dimensional input-based research methods. Two-dimensional input methods consist of traditional machine learning model and two-dimensional deep learning models. The three-dimensional input method basically takes the three-dimensional deep learning model as the main backbone. Due to the abundant spatial pathological information in medical examination images, three-dimensional methods generally have more advantages in the detection effect. In addition, some researchers have studied the characteristics of small sample size of medical test images, or introduced different types of data to establish a multi-modal fusion algorithm.

Rieke et al. trained on a 3D CNN model and applied four gradient-based and occlusion-based approaches to visualization, promoting clinical impact and trust in computer-based decision support systems (Rieke et al., 2018). But 3DCNN contains the risk of network degradation and gradient disappearance/gradient explosion after increasing the number of middle layers. Rieke et al. trained on a 3D CNN model and applied four gradient-based and occlusion-based approaches to visualization, promoting clinical impact and trust in computer-based decision support systems. But 3DCNN contains the risk of network degradation and gradient disappearance/gradient explosion after increasing the number of middle layers. Based on convolutional autoencoder (CAE), Kanghan et al. proposed supervised and unsupervised classification methods for the diagnosis of Alzheimer's disease. The combination of convolutional layer and pooling layer of CAE is relatively fixed, which limits to construct more complex network structure. Guan et al. constructed a preliminary standardized model framework based on ResNet, VGG, DenseNet and other networks, and comprehensively tested and compared these models using standard MRI image data sets of Alzheimer's disease. They found that these simple architectures performed similarly on the task, and the pre-training process of these methods has less impact on accuracy. Korolev et al. proposed VoxCNN based on ResNet to classify MRI images. This model can achieve better performance using a small training dataset, and be applied to 3D MRI images without the need of intermediate handcrafted feature extraction. However, VoxCNN includes the module of 3D-Resnet, which needs to consume more training time and more computing resources in training.

In the above reports, these methods have achieved excellent performance in their selected datasets. However, these studies lacked comparability and robustness among themselves. Most studies needs lots of GPU resources to train the model, which makes it difficult to apply the research results widely.

## 3. Methods

### 3.1. Approvement statement of institutional review board

This study is approved by institutional board with written informed consent waived. All experiments including any relevant details are approved by institutional and/or licensing committee. All experiments on humans and/or the use of human tissue samples were performed in accordance with relevant guidelines and regulations. All experimental protocols were approved by the Steering Committee of Alzheimer's Disease Neuroimaging Initiative and Academic Committee of Jinan University. Informed consent was obtained from all subjects and/or their legal guardian(s).

### 3.2. Data acquisition

All MRI image data used in this study were obtained from the Alzheimer's Disease Neuroimaging Initiative (ADNI) database (http://adni.loni.usc.edu/) (Petersen et al., 2010). Founded in 2003, ADNI is a public-private partnership led by Principal Investigator Michael W.Weiner, MD. The primary goal of ADNI is to test whether serial magnetic resonance imaging (MRI), positron emission tomography (PET), other biomarkers, and clinical and neuropsychological assessments can be combined to measure the progression of mild cognitive impairment (MCI) and early Alzheimer's disease (AD). The latest information about the ANDI database can be found at (http://adni.loni.usc.edu/). The ADNI database consists of four sub-databases, including ADNI-1, ADNI-Go, ADNI-2, and ADNI-3, which are interdependent. The diagnostic labels of these medical image data are given by doctors after a series of tests.

**Figure 1:**
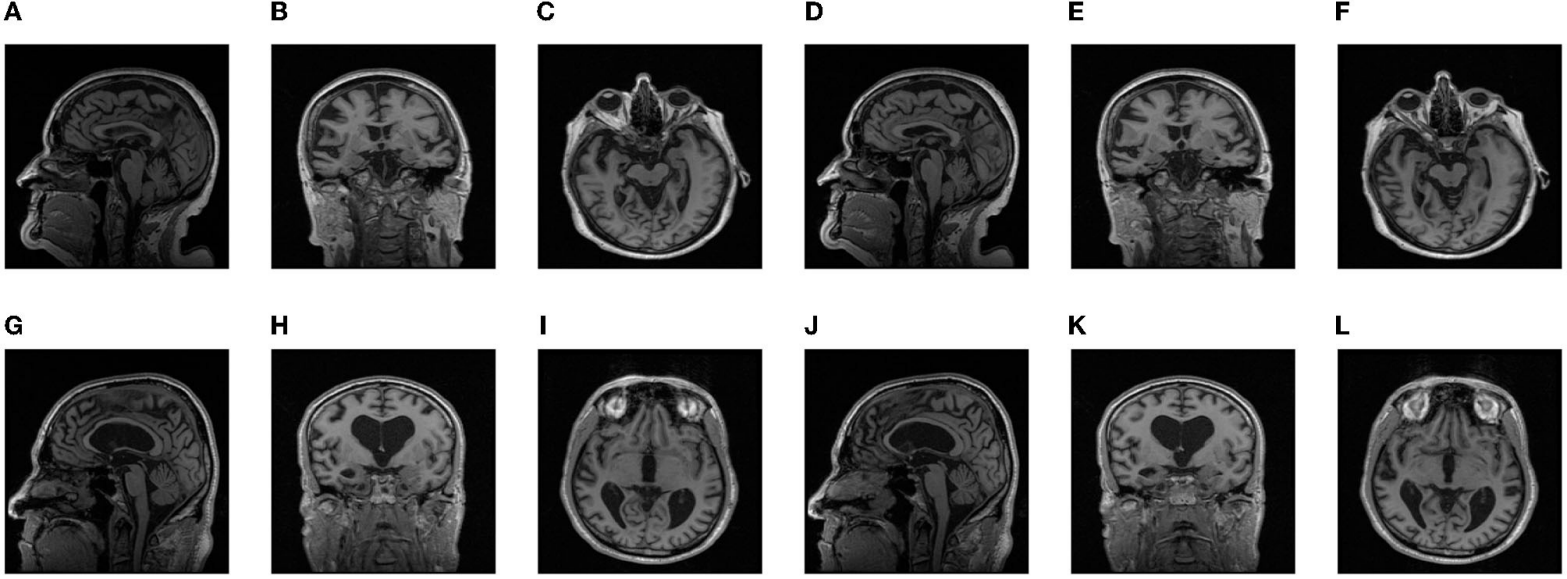
Partial slice samples of some ADNI datasets: **(A–F)** AD, **(G–L)** NC.

ADNI provides several standardized datasets for researchers to study. In our research, ADNI1 Complete 2Yr 3T standardized dataset is chosen to train our model, including scans of patients taken at 6, 12, 18, and 24 months after diagnosis. The dataset contains 434 subjects, including 77 of AD, 206 of MCI, and 151 of NC. The demographic information of the dataset is shown in Table 1. We searched the ADNI1 Complete 2Yr 3T standardized dataset in the ADNI database, packaged it and downloaded. All image data in this study were stored in NifTI format. Figure 1 shows the slides samples of this dataset.

**Table 1:** The structure of residual Conv module used in feature mapping layer and enhancement layer.

**Layer name**	**Number of Bottle Neck block**	**Number of kernel**	**Kernel size**	**Input Size**	**Output size**
3D Conv Layer	0	64	7×7×7	224×224×128	224×112×64
3D AvgPool Layer	0	64	3×3×3	224×112×64	112×56×32
3D Residual Module 1	3	256	1×1×1	112×56×32	112×56×32
			3×3×3		
			1×1×1		
Global AvgPool	0	256	0	112×56×32	256
3D Residual Module 2	4	512	1×1×1	112×56×32	56×28×16
			3×3×3		
			1×1×1		
Global AvgPool	0	512	0	56×28×16	512

### 3.3. Data preprocessing

Since the data came from many different patient samples, the size of different data and the location of key parts in the image may vary to some extent, while the neural network model required that the size of each input be consistent. At the same time, due to its intensity and other attributes, MRI image is not suitable to be directly used as the input image of the model, so it needs to be transformed to some extent. In conclusion, we have to take a series of pre-processing measures for the data, so that it can be converted into appropriate input data, and is conducive to improving the performance of the model.

In this research, The primitive image size of our ADNI dataset was 256×256×160. Firstly, Since the pixel size of different medical scanned images is not the same, all pixels need to be resampled at a fixed homogeneous resolution. We resampled all MRI images to 1.5-mm isotropic voxels. Then, we scaled the intensity of the images to the range (0, 1). Since the region of interest (ROI), namely the patient's brain, was basically concentrated in the center of the image, we cropped the image based on the central region and removed some peripheral background areas. The final output after processing were 224×224×128-pixel grid resulting in 336×336×192-*mm*^2^ volume. The above data preprocessing operations were based on Python 3.8 environment, using package Monai (https://monai.io/) and package Numpy (https://numpy.org/) for processing.

### 3.4. Model details and training

#### 3.4.1. Broad learning system

Although traditional deep neural networks show good accuracy in traditional recognition and classification tasks, they also expose problems such as large number of hyperparameters and long time consuming. At the same time, with the publication of more types of datasets, researchers need to seek a new method with simple structure and fast operation to deal with different requirements and tasks. In studies over the past few years, classical single-layer network structures such as Extreme Learning Machine (ELM) (Huang et al., 2006) and Random Vector Functional Link Neural Network (RVFLNN) (Pao et al., 1994) have been proposed successively.

On the basis of RVFLNN (Pao et al., 1994), Chen et al. proposed Broad Learning System (BLS) (Chen and Liu, 2017a,b). In the structure of BLS, the basic linear feature of the input was extracted by the feature mapping layer. The further feature of the former layer was extracted by the enhancement layer which contained a non-linear function. Then, the output of these layers were concat together and transferred to the output layer for classifying. Since there is only two layers of structure, BLS does not need to calculate a large number of weight parameters for multiple middle layers, which saving a lot of calculation resources and reducing the training time of the model. Previous experimental results demonstrate that BLS can still achieve excellent performance in the basic test of image recognition, which proves that BLS has good potential in the field of computer vision (Chen and Liu, 2017a,b).

For a given input sample ***X∈******R***^***n×m***^, where ***n*** represents the number of samples, ***m*** represents the feature dimension of the sample. The feature mapping layer is composed of the combination of feature nodes. The feature nodes and feature mapping layer of broad learning system can be expressed as following:


(1) $$\begin{array}{l} d_{i}=\varphi_{i}\left(XW_{e_{n}}+\beta_{e_{n}}\right) \end{array}$$

(2) $$\begin{array}{l} D^{n}=\left[d_{1},d_{2},......d_{n}\right] \end{array}$$

where **φ***_**i**_* is the selectable linear or non-linear activation function, ***W***_***e***_***n***__ is the random weight, and ***W***_***e***_***n***__ is the random bias. ***W***_***e***_***n***__ and **β***_**e**_**n**__* are usually optimized by sparse auto-coding algorithm. The enhancement node and enhancement layer of BLS can be denoted as:


(3) $$\begin{array}{l} e_{j}=\delta_{j}\left(D^{n}W_{h_{m}}+\beta_{h_{m}}\right) \end{array}$$

(4) $$\begin{array}{l} E^{m}=\left[E_{1},E_{2},......E_{m}\right] \end{array}$$

where **δ***_**j**_* is the non-linear activation function, ***W***_***h***_***m***__ is the random weight, and **β***_**h**_**m**__* is the random bias. Then, the feature mapping layer and the enhancement layer are concated and transferred to the output layer. Since ***W***_***e***_***n***__, ***W***_***e***_***n***__, ***W***_***h***_***m***__, and **β***_**h**_**m**__* remain unchanged in the training process, the objective function of BLS is:


(5) $${\;}_{W}(||(Y-Y^{\prime })|{|}_{2}^{2}+\frac{\lambda }{2}||W|{|}_{2}^{2})$$

where ***W*** is the weight of the output layer of the BLS, ***Y*** is the label of ***X***, $||(Y-Y^{\prime })|{|}_{2}^{2}$ is used to control the minimization of training error, $\frac{\lambda }{2}||W|{|}_{2}^{2}$ is used to prevent model overfitting, and ***λ*** is the regularization coefficient. Then, ***W*** can be obtained by seeking the pseudo-inverse of ridge regression:


(6) $$\begin{array}{l} W=G^{+}Y \end{array}$$

(7) $$\begin{array}{ll} G^{+}=\underset{\lambda \to 0}{\lim}{\left(\lambda I+G^{T}G\right)}^{-1}G^{T} & \;\;\;\;(7) \end{array}$$

where ***I*** is the identity matrix. Through the above steps, we constructed a complete Broad Learning System.

#### 3.4.2. Deep-broad ensemble model

Three-dimension radiographic images contain complex pathological spatial information. However, the original broad learning system can only receive two-dimensional features as input, and the ability to extract complex image features is weak. Integrating the deep convolution module can effectively improve the feature extraction ability of the broad learning system, which can better the performance for its classification and recognition (Chen et al., 2018).

In this paper, we proposed a deep-broad ensemble model for early recognition of Alzheimer's disease based on the above ideas, which aims to maintain a considerable performance of classification, reduce the dependence of hardware and improve the efficiency of the model.

**Figure 2:**
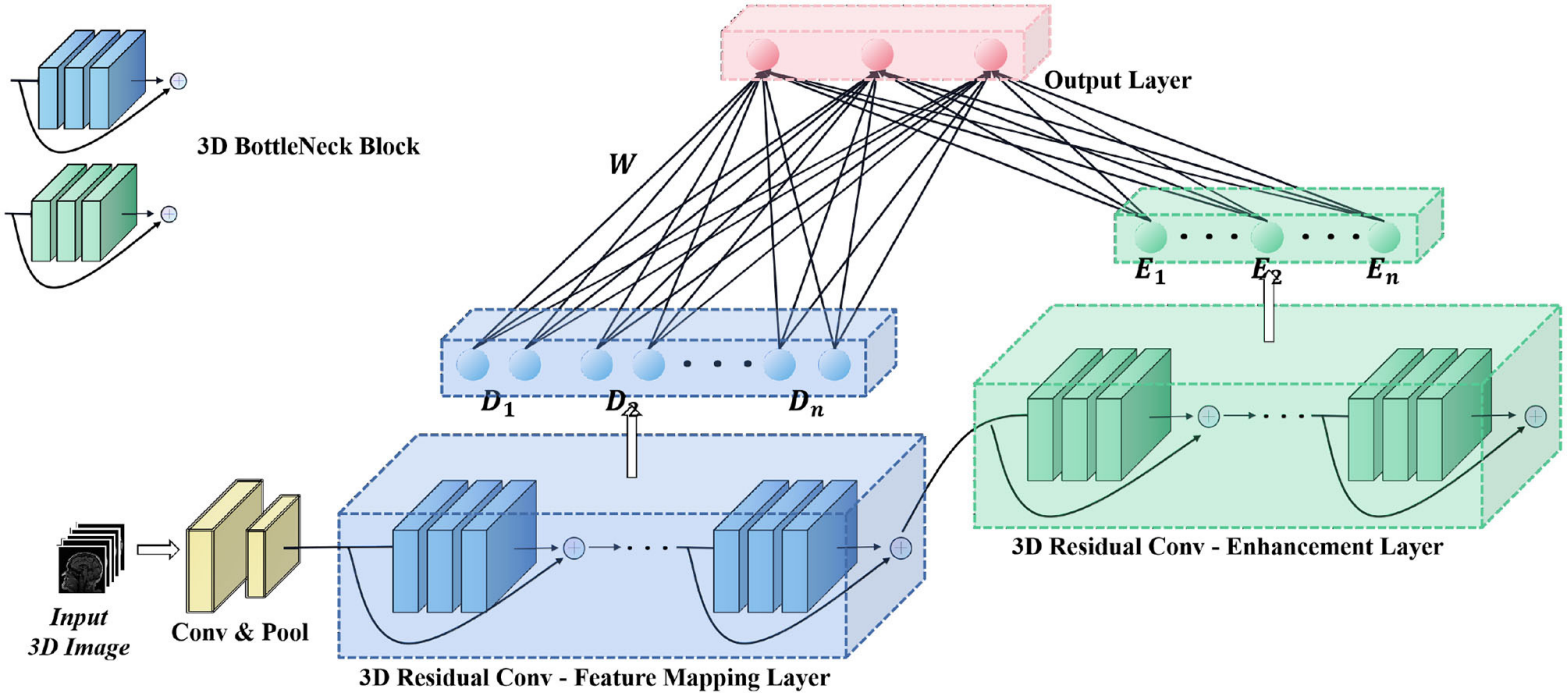
Structure of proposed deep-broad ensemble model.

As shown in Figure 2, we used a convolution-pooling layer to initially extract the features of the original input. The size of the convolution kernel used was 7×7×7, and the size of the pooling module was 3×3×3.The backbone of the model is composed of a 3D residual convolution—feature mapping layer and a 3D residual convolution—enhancement layer. The 3D residual convolution—feature mapping layer can be divided into residual convolution module and feature mapping module. The former is composed of several 3D bottleneck convolution modules (He et al., 2016), which are used to extract shallow features of the input, and transform these features into feature vectors with a size of 256 through the global pooling, and then input into the feature mapping module for further processing; The 3D residual convolution enhancement layer also includes several 3D bottleneck convolution modules as the former to extract deeper features. The feature vectors are then input to the enhancement module through the global pooling, which is different from the input of the enhancement layer in the original BLS. Detailed parameters of the deep module used in the whole backbone model are shown in Table 1. Finally, feature mapping module and enhancement module are mapped to the output layer to produce classification results.

For a Given the original MRI image input ***X***, the output feature vector after the first convolution-pooling layer is:


(8) $$\begin{array}{l} X_{base}=\lambda_{conv-pool}\left(X\right) \end{array}$$

The feature vector of the output after the residual convolution module of the 3D residual convolution-feature mapping layer and the 3D residual convolution-enhancement layer can be denoted as:


(9) $$\begin{array}{l} X_{d}=\lambda_{d}\left(X_{base}\right) \end{array}$$

(10) $$\begin{array}{l} X_{e}=\lambda_{e}\left(\lambda_{d}\left(X_{base}\right)\right) \end{array}$$

where λ_*d*_() indicates the residual convolution module of 3D residual convolution-feature mapping layer, and λ_*e*_() indicates the residual convolution module of 3D residual convolution-enhancement layer. According to formula (1)–(4), feature nodes, feature mapping modules, enhancement nodes, and enhancement mapping modules of Broad learning can be denoted as:


(11) $$\begin{array}{l} d_{i}=\left[\varphi_{i}\left(X_{d}W_{e_{n}}+\beta_{e_{n}}\right)\right] \end{array}$$

(12) $$\begin{array}{l} D^{n}=\left[d_{1},d_{2},......d_{n}\right] \end{array}$$

(13) $$\begin{array}{l} e_{j}=\left[\delta_{j}\left(X_{e}W_{h_{m}}+\beta_{h_{m}}\right)\right] \end{array}$$

(14) $$\begin{array}{l} E^{m}=\left[E_{1},E_{2},......E_{m}\right] \end{array}$$

According to formula (6)–(7), the final classification output ***Y*** can be obtained. Thus, we constructed a 3D Convolution Broad Learning System based on 3D medical image input.

#### 3.4.3. Model training

After data preprocessing, the original image was converted into a 224×224×128 matrix as the input of the model. The server we used for model training had a AMD EPYC 7543 32-core Processor, 90 GB of RAM, Nvidia GeForce RTX 3090 GPU with CUDA 11.1.

We set up two groups of experiments whose convolution module was non-pre-trained and pre-trained respectively for comparison. The dataset used for pre-training was ADNI training dataset (347 images). In the pre-training experiment, we set cross entropy as the loss function, with the learning rate of 0.0001, the optimizer of momentum-SGD, and the batch size of 4.

For the broad module in the model, the range of feature nodes was [500–4,000], the range of enhancement nodes is [100–1,000], and the range of sparsity coefficient is [0.4–0.7]. We used Pytorch 1.8 and Numpy to construct the program of the model presented in this article, and all programs and experiments were run in Python 3.8.

### 3.5. Model testing and analysis

For the three groups of models after training, we used the ADNI test dataset for testing. The model finally outputs the probability that an image belongs to one of these categories. The category with the highest probability was selected as the classification result. We calculated the final classification accuracy, Precision and Recall based on this result. In addition, We studied the stability of the model by modifying the hyperparameters.

### 3.6. Clinical interpretation of MRI

To compare the performance of our proposed model with that of an actual radiology reader, a board-certified nuclear medicine physician with several years of experience (HuanHua Wu, nuclear medicine) was invited to perform a discriminative analysis of 87 MRI images from the ADNI test dataset. In order to prevent data leakage, the reader can only obtain MRI image data and the number of the subject, and analyze them based on their professional experience. We will calculate the corresponding indicators based on this result.

## 4. Results

### 4.1. Demographics

As shown in Table 2, The dataset used in this study contained 434 MRI images from 86 patients, which contained three types of Alzheimer's symptoms: AD, patients with Alzheimer's disease; MCI, mild cognitive impairment; NC, normal person. Seventy-seven images were obtained from AD, 151 from NC, and 206 from MCI. Partial slice images of AD and NC cases in the dataset are shown in **Figure 4**. The average age of all patients was 75.44 years old (range from 55 to 90 years), including 73.58 years old for female (range from 55 to 90 years) and 77.20 for male (range from 57 to 89 years). The average age of AD groups was 75.32 years (range from 57 to 90 years), with the average age of 75.04 years for female (range from 64 to 90 years) and 75.71 years for male (range from 57 to 87 years). The average age of MCI groups was 74.62 years (range from 55 to 89 years), with the average age of 69.56 years for female (range from 55 to 82 years) and 77.45 years for male (range from 63 to 89 years). The average age of NC groups was 76.62 years (range from 70 to 88 years), with the average age of 76.08 years for female (range from 71 to 82 years) and 77.46 years for male (range from 70 to 88 years).

**Table 2:** Demographics of ADNI dataset.

**Diagnosis**	**Number of patients**	**Number of images**	**Gender Male/Female**	**Average age All [Male/Female]**
AD	18	77	32/45	75.32 [75.71/75.04]
NC	33	151	58/93	76.62 [77.46/76.08]
MCI	35	206	132/74	74.62 [77.45/69.56]
Total	86	434	222/212	75.44 [77.20/73.58]

### 4.2. Result of training

The preprocessed dataset was divided into training set and test set in a ratio of 0.7:0.3. We trained models for AD/CN, AD/MCI, and MCI/NC tasks respectively. Accuracy (ACC), sensitivity (SEN), and F1-score were used to evaluate the performance of them, and the training time of each model was recorded.

**Figure 3:**
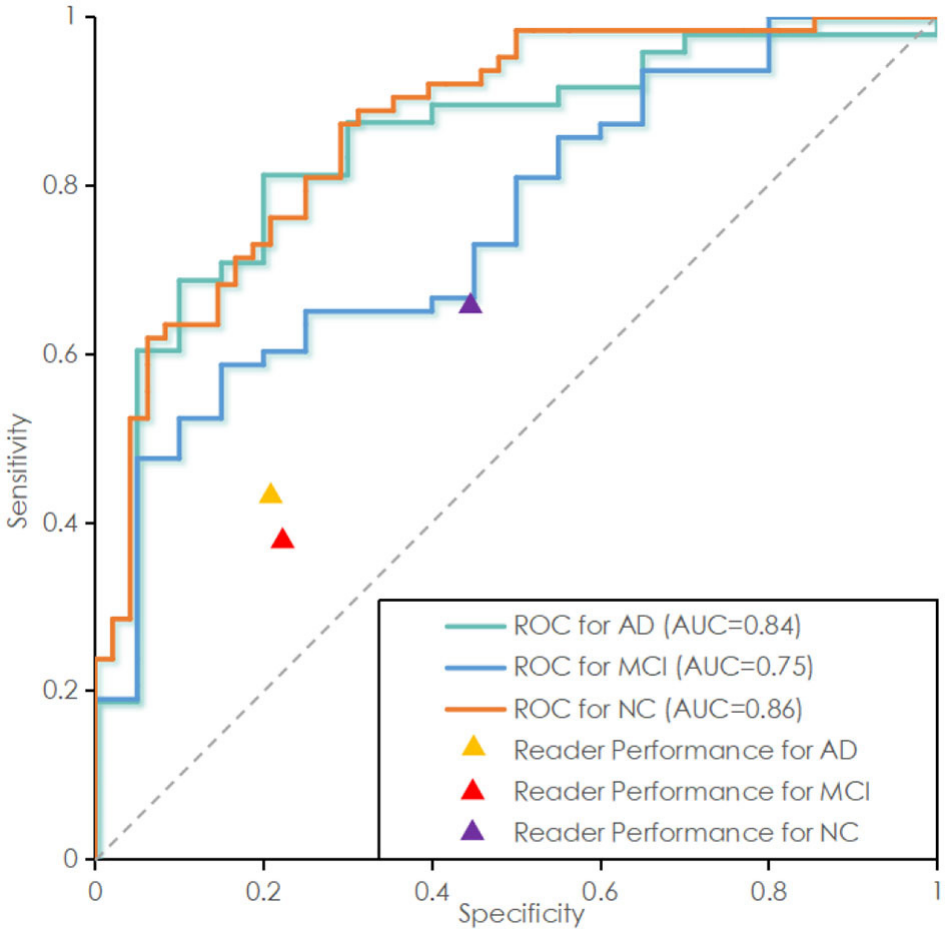
Receiver operating characteristic (ROC) curve of the proposed deep-broad ensemble model trained on 70% of 3YR 2T ADNI dataset and tested on the remaining 30% of ADNI dataset and independent test set. ROC curve labeled Alzheimer Disease (AD) represents the essential performance for distinguishing AD vs. all other cases. ROC curves for Mild Cognitive Impairment (MCI) and Normal Control (NC) are also reported for technical completeness.

As shown in **Table 4**, in the tests of AD/CN, AD/MCI, and MCI/NC, the average of accuracy for prediction were 90.97, 91.16, and 83.39%. SEN is 91, 94, 85%. F1-score is 91, 94, 84%. The above results indicated that the proposed model has good discrimination ability in AD/NC and AD/MCI tasks, but weak discrimination ability in MCI/NC tasks. The ROC curves of the proposed deep-broad ensemble model method trained on 70% of ADNI dataset were shown in Figure 3.

**Figure 4:**
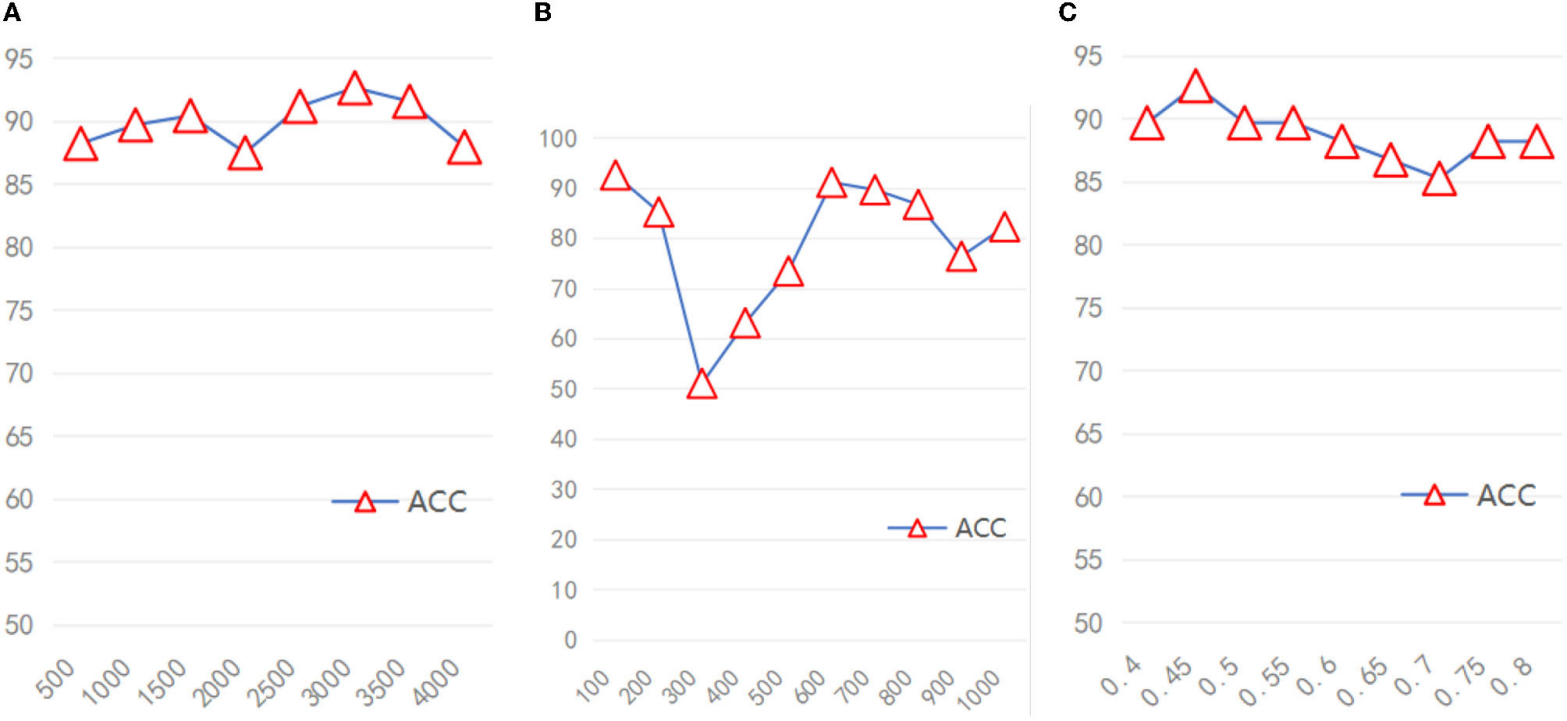
The accuracy of the model under different hyperparameters **(A)** Feature nodes, **(B)** Enhancement nodes, **(C)** Sparsity coefficient.

Because the broad module of the model required different hyperparameters, We also verified the stability of the model based on different hyperparameters. Due to the huge range of hyperparameters (range of feature mapping nodes: 500–4,000, range of enhancement node: 100–1,000, range of sparsity coefficient: 0.4–0.7), we took several hyperparameters values as representative. As shown in Figures 4A–C, when the number of feature mapping nodes and the sparse coefficient increased, the model maintained good stability. When the number of enhancement nodes increases, the stability of the model is generally acceptable, excluding some unstable intervals.

In addition, We explored the influence of the pre-training for the deep module on the final classification performance of the model. As shown in Table 3, the non-pre-trained model performed better than the pre-trained model on the AD/NC and NC/MCI tasks (maximum accuracy of 92.65/90.57 and 84.68/76.44), while the two performed similarly on the AD/MCI tasks (maximum accuracy of 92.76/91.57).

**Table 3:** Comparison of the proposed model and radiology readers.

**Method**	**Accuracy (%)**	**Sensitivity (%)**	**Precision (%)**
AD vs. NC			
Deep-broad ensemble model (Pre-trained)	90.57	91	91
Deep-broad ensemble model (Not Pre-trained)	92.65	91	91
AD vs. MCI			
Deep-broad ensemble model (Pre-trained)	93.58	92	91
Deep-broad ensemble model (Not Pre-trained)	91.57	92	92
MCI vs. NC			
Deep-broad ensemble model (Pre-trained)	76.44	74	75
Deep-broad ensemble model (Not Pre-trained)	84.68	85	84

The data are presented as Maximum.

Since the BLS itself had considerable ability of feature fitting, the deep module of the proposed model was mainly used to further extract complex space features of medical image, enhancing the feature extraction ability of BLS. Therefore, the pre-training of deep module was not decisive. The broad module is decisive in the fitting of image features. If the cost of pre-training process was removed, the training time of the model proposed can be further reduced, lowing the dependency of hardwares and improving the efficiency of the model.

### 4.3. Model interpretation: t-SNE plot

**Figure 5:**
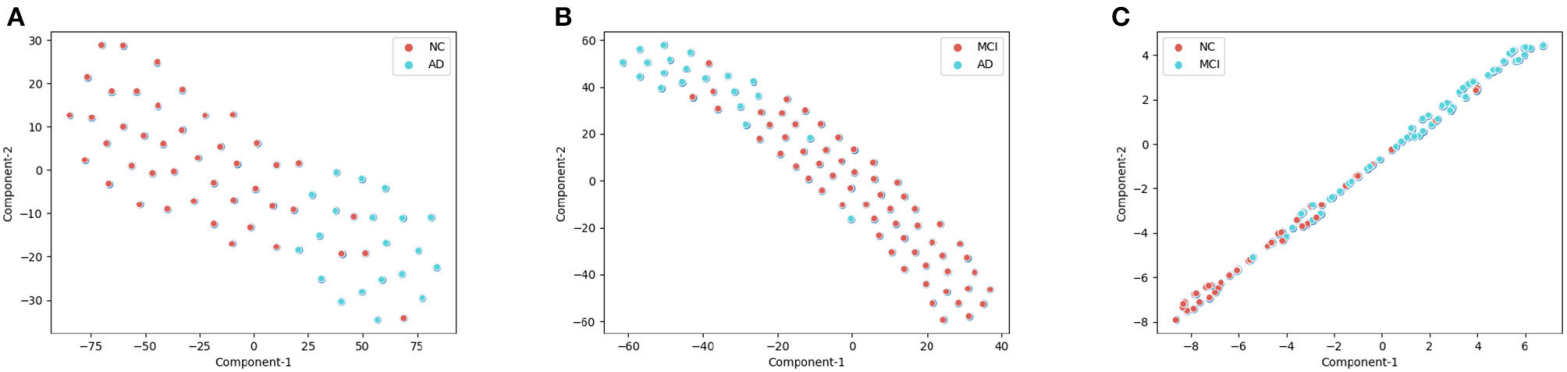
The visualization of training set after dimension reduction with t-distributed stochastic neighbor embedding (t-SNE). Each point represents the final features of the proposed deep-broad ensemble model. **(A)** t-SNE for the AD/CN, **(B)** t-SNE for the AD/MCI, **(C)** t-SNE for the NC/MCI.

As shown in the Figure 5, We clustered the final features of the models in the three experiments respectively after dimension reduction by t-SNE. In AD/NC and AD/MCI classification experiments, the corresponding categories were almost pure, with only a small amount of mixing. In the NC/MCI experiment, the mixture of the two categories was more common. Therefore, we concluded that the proposed model is highly sensitive to AD categories, because most of the sample points were in the clustering of AD; we achieved high accuracy in both experiments.

### 4.4. Comparison to previous works

We compared the proposed model with some previous works, which had developed several deep models in this task. Due to the lack of relevant hyperparameter reference, we set the number of training epochs to 40 for each work. As shown in Table 4, in the three tasks of AD/CN, AD/MCI and MCI/NC, the accuracy of the proposed model outperformed these works. The training time of the proposed model was much shorter than that of these deep models, because there was no need to update the weight parameters of the deep module of the proposed model. Therefore, compared with the previous works, the proposed model had a considerable optimization effect, less dependence on computer hardware, and was easier to deploy in the actual diagnosis process.

**Table 4:** Comparison of the proposed model and previous related work.

**Method**	**Accuracy (%)**	**Sensitivity (%)**	**F1-score(%)**	**Time(s)**
AD vs. NC				
Deep-broad ensemble model	90.97 ± 1.02	91	91	1.3731
ResNet-18 (He et al., 2016)	73.53 ± 0.97	74	67	^†^1920
DenseNet-121 (Huang et al., 2017)	69.12 ± 2.93	69	69	^†^2480
VCNet (Rieke et al., 2018)	70.56 ± 2.91	71	58	^†^1440
CAE (Oh et al., 2019)	85.24 ± 3.97	88	nan	nan
ICAE (Oh et al., 2019)	86.60 ± 3.66	88	nan	nan
Guan's work (Guan et al., 2019)	69.12 ± 0.57	69	64	^†^2120
VoxCNN (Korolev et al., 2017)	72.06 ± 1.43	72	64	^†^2800
AD vs. MCI				
Deep-broad ensemble model	91.16 ± 3.86	94	94	1.4745
ResNet-18 (He et al., 2016)	77.11 ± 1.20	77	68	^†^1920
DenseNet-121 (Huang et al., 2017)	70.59 ± 1.47	71	58	^†^2480
VCNet (Rieke et al., 2018)	74.69 ± 1.24	75	65	^†^1440
CAE (Oh et al., 2019)	74.68 ± 6.04	75	nan	nan
ICAE (Oh et al., 2019)	75.06 ± 3.86	77	nan	nan
Guan's work (Guan et al., 2019)	71.08 ± 2.41	71	67	^†^2120
VoxCNN (Korolev et al., 2017)	75.90 ± 0.10	76	66	^†^2800
MCI vs. NC				
deep-broad ensemble model	83.39 ± 1.31	85	84	1.6825
ResNet-18 (He et al., 2016)	57.65 ± 0.90	58	55	^†^1920
DenseNet-121 (Huang et al., 2017)	55.86 ± 2.81	56	45	^†^2480
VCNet (Rieke et al., 2018)	58.56 ± 0.91	59	53	^†^1440
CAE (Oh et al., 2019)	62.83 ± 5.17	66	nan	nan
ICAE (Oh et al., 2019)	63.34 ± 4.16	69	nan	nan
Guan's work (Guan et al., 2019)	56.76 ± 3.72	58	57	^†^2120
VoxCNN (Korolev et al., 2017)	59.46 ± 0.90	59	49	^†^2800

Unless otherwise stated, the data are presented as Mean ± Std.

† The data here is represented as the average of the three tasks.

### 4.5. Comparison to clinical Interpretations

As shown in Table 5, in the above tasks, the accuracy of radiology readers were 68.57, 75.00, 61.76%. Sensitivity were 38, 43, 66%. F1-score were 35, 29, 68%. Compared to radiology reader's work, the proposed model had better performance in the detection of ADNI datasets, which has statistical significance.

**Table 5:** Comparison of the proposed model and radiology readers.

**Method**	**Accuracy (%)**	**Sensitivity (%)**	**Specificity**	**F1-score(%)**
AD vs. NC				
Deep-broad ensemble model	92.65	91	89	91
Radiology readers	68.57	38	22	35
AD vs. MCI				
deep-broad ensemble model	93.58	94	92	94
Radiology readers	75.00	43	21	29
MCI vs. NC				
deep-broad ensemble model	84.68	85	81	84
Radiology readers	61.76	66	45	68

The data are presented as Maximum.

## 5. Discussion

The diagnosis and treatment of Alzheimer's disease is becoming an important medical issue for decades to come. Millions of Patients with Although Alzheimer's disease provides a rich data base for the improvement of diagnostic theories, it brings great work pressure and challenges to front-line doctors.

At present, computer science researchers had developed many detection models for radiographic images of the Alzheimer's disease. However, these current models almost consisted of single deep networks. This would lead to the problem that the models were highly dependent on hardwares, which was difficult to be popularized in non-urban areas where relevant hardware was lacking. To solve the above problems, we constructed a deep-broad ensemble model for radiographic images based on the novel BLS which has higher efficiency. Then, we trained and tested the model using the MRI dataset obtained from ADNI database, and calculated the corresponding accuracy, sensitivity and F1-score according to the results. We also compared the model with some previous work and results from radiology reader. The results demonstrates that compared with the previous work and the reader, the proposed model has better performance and greatly reduces the training time. Meanwhile, we studied the effect of the deep convolution module and the improved BLS module on the model. The results demonstrates that BLS was still the core of the model. The function of the deep module was to enhance the feature extraction capability of the BLS module, thus requiring no pre-training, which would greatly improve the performance of the model proposed in this paper. Our experiment had certain limitations. Firstly, the amount of data used in this study is still relatively small (434 images) due to the limited number of public medical image datasets for Alzheimer's disease currently available for research. Therefore, the robustness of the proposed model has not been verified on larger and more general data, which limits the application of our proposed model in real scenarios.

Second, BLS is a non-deep learning framework. Although its interpretability of it has been proven (Chen et al., 2018), BLS is not as widely used as deep neural network. Broad Learning System itself also has the limitation of relatively low accuracy, and its application in medical imaging and other fields lacks of universal reference. The hyperparameter setting of the proposed model relies on the previous research experience of machine learning researchers and lacks a better adjustment method (Gong et al., 2021).

In general, our experiment and research results demonstrate that our proposed deep-broad ensemble model method significantly reduces the training time while maintaining good detection performance. This makes our model play a referential role in practical medical image diagnosis and reduces the dependence on external hardware. With the opening of more medical image data, the model proposed in this paper can be better applied to first-line clinical diagnosis and provide reliable reference for doctors and medical image readers.

## Data availability statement

The original contributions presented in the study are included in the article/supplementary material, further inquiries can be directed to the corresponding author/s.

## Ethics statement

The studies involving human participants were reviewed and approved by Academic Committee of Jinan University. The patients/participants provided their written informed consent to participate in this study. Written informed consent was obtained from the individual(s) for the publication of any potentially identifiable images or data included in this article.

## Author contributions

PM: conceptualization, methodology, validation, visualization, software, writing—original draft, review, and editing, and project administration. JW: supervision and project administration. ZZ: supervision and writing—review and editing. CC: conceptualization, resources, and supervision. Alzheimer's Disease Neuroimaging Initiative: data provision. JD: conceptualization, methodology, validation, writing—review and editing, supervision, project administration, and funding acquisition. All authors contributed to the article and approved the submitted version.
